# Emergence of synaptic organization and computation in dendrites

**DOI:** 10.1515/nf-2021-0031

**Published:** 2022-02-23

**Authors:** Jan H. Kirchner, Julijana Gjorgjieva

**Affiliations:** Computation in Neural Circuits Group, Max Planck Institute for Brain Research, Max-von-Laue-Str. 4, 60438 Frankfurt, Germany; Technical University of Munich, School of Life Sciences, 85354 Freising, Germany

**Keywords:** cortex, dendrite, development, organization, synaptic plasticity, Kortex, Dendrit, Entwicklung, Organisation, synaptische Plastizität

## Abstract

Single neurons in the brain exhibit astounding computational capabilities, which gradually emerge throughout development and enable them to become integrated into complex neural circuits. These capabilities derive in part from the precise arrangement of synaptic inputs on the neurons’ dendrites. While the full computational benefits of this arrangement are still unknown, a picture emerges in which synapses organize according to their functional properties across multiple spatial scales. In particular, on the local scale (tens of microns), excitatory synaptic inputs tend to form clusters according to their functional similarity, whereas on the scale of individual dendrites or the entire tree, synaptic inputs exhibit dendritic maps where excitatory synapse function varies smoothly with location on the tree. The development of this organization is supported by inhibitory synapses, which are carefully interleaved with excitatory synapses and can flexibly modulate activity and plasticity of excitatory synapses. Here, we summarize recent experimental and theoretical research on the developmental emergence of this synaptic organization and its impact on neural computations.

## Zusammenfassung

Einzelne Neuronen im Gehirn weisen erstaunliche Rechenfähigkeiten auf, die sich im Laufe der Entwicklung allmählich entwickeln und es den Neuronen ermöglichen, in komplexe neuronale Schaltkreise integriert zu werden. Diese Fähigkeiten leiten sich unter anderem von der genauen Anordnung der Synapsen auf den Dendriten der Neuronen ab. Während der genaue rechnerische Nutzen dieser Anordnung noch unbekannt ist, zeichnet sich ein Bild ab, in dem Synapsen nach ihren funktionellen Eigenschaften über mehrere räumliche Ebenen hinweg organisiert sind. Während auf der lokalen Ebene (zehn Mikrometer) exzitatorische synaptische Eingänge dazu neigen, Cluster entsprechend ihrer funktionalen Ähnlichkeit zu bilden, zeigen synaptische Eingänge auf der Ebene einzelner Dendriten oder des gesamten Baums “dendritic maps”, bei denen die Funktion exzitatorischer Synapsen gleichmäßig mit der Position auf dem Dendriten variiert. Die Entwicklung dieser Organisation wird durch inhibitorische Synapsen unterstützt, die präzise mit exzitatorischen Synapsen verflochten sind und die Aktivität und Plastizität exzitatorischer Synapsen flexibel modulieren können. Hier fassen wir aktuelle experimentelle und theoretische Forschung über die Entstehung dieser synaptischen Organisation in der Entwicklung und ihren Einfluss auf neuronale Berechnungen zusammen.

## Introduction

Neurons process information in the form of electrical signals called action potentials. These signals are transmitted via synapses from one neuron to another. At a synapse, an electrical signal induces the release of neurotransmitters which affect the receiving neuron’s membrane potential. The majority of synapses are found on dendrites, branch-like extensions of a neuron that receive electrical stimulation from other neurons and carry it to the neuron’s cell body called the soma. Depending on the neurotransmitter a synapse releases, a synapse is either excitatory, i.e., usually depolarizing the membrane potential via the release of acetylcholine or glutamate, or inhibitory, i.e., usually hyperpolarizing the membrane potential via the release of gamma-aminobutyric acid (GABA) or glycine. The exact arrangement of excitatory and inhibitory synapses influences the generation of action potentials at the soma, and hence the transmission of information to other neurons, and is of central importance for information processing in the brain (Stuart et al., 2016).

During early development, various single neuron and neural circuit properties, such as the organization of synapses, emerge through the interaction of multiple factors, including genetically regulated cell specification and activity-dependent circuit formation and refinement. Since many of the sensory organs in the developing brain are immature, much of the early neural activity is generated *spontaneously* in the absence of sensory stimulation. Almost all neural circuits in the developing brain can generate spontaneous activity, including the sensory cortex on which we focus here (Leighton and Lohmann, 2016). Spontaneous activity is usually highly structured and contains spatiotemporal correlations to instruct the formation, the removal, and the changes in strength of synaptic inputs. The developing retina exhibits one of the best studied examples of spontaneous activity known as retinal waves, which directly influence connectivity refinement and receptive field tuning in downstream visual areas such as the superior colliculus and the thalamus (Blankenship and Feller, 2010). Specific activity-dependent plasticity mechanisms drive this connectivity refinement (Richter and Gjorgjieva, 2017). The exact form of these plasticity mechanisms and their functional implications are an active area of research (Agnes and Vogels, 2021; Hiratani and Fukai, 2018; Kirchner and Gjorgjieva, 2021; Mikulasch et al., 2021; Sezener et al., 2021).

There are different computational and theoretical approaches to investigate the emergence of organization in the developing brain. In one approach, neurons are modeled as so-called *point neurons* that conceptualize neurons as single points without spatial extent. Often, point neurons are assumed to directly integrate synaptic inputs and transform them into spiking outputs while ignoring the transformations implemented by dendrites. Point neurons have the advantage that they are amenable to mathematical analysis while still capturing the ability of the neuron to generate action potentials and can be easily connected when simulating large networks. At the other end of the spectrum, multicompartment neuron models include fully reconstructed dendrites to carefully incorporate the influence on the soma of individual synaptic inputs across the dendritic tree. Multicompartment models are often equipped with a variety of ion channels, allowing them to produce a wide range of local, nonlinear transformations of the input. While these models are typically mathematically intractable, they reveal the profound impact of synapses’ distribution on dendritic, cellular, and network computations and neural information processing more generally (Poirazi et al., 2003).

Here, we adopt a third perspective, similar to the passive multicompartment model, in which we retain the dendrite’s shape while abstracting away many details of a full biophysical model. We distinguish between different spatial scales of synaptic organization on a dendrite, local and global, and summarize recent experimental and theoretical progress on understanding the properties, functions, and developmental emergence of this organization. We highlight differences and commonalities between excitatory and inhibitory synapse organization and possible functional consequences of their interaction. Finally, based on experimental data from the developing retina, we propose a model for the developmental emergence of balanced excitation and inhibition.

## Organization of excitatory synapses

**Figure 1:**
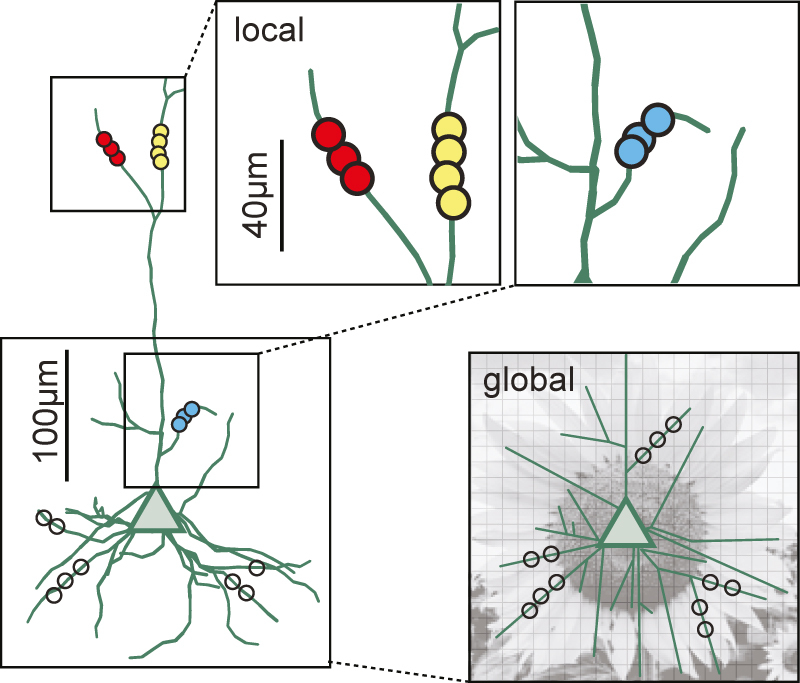
A schematic of a dendrite with synapses (colored dots). Synapses exhibit organization at multiple spatial scales. At the local scale of tens of microns (top right), synapses form clusters in which synapses with similar selectivity (indicated by the color) are in spatial proximity. At the global scale (bottom right), synapses form dendritic maps, where nearby locations in visual space, represented by the sunflower, are encoded by synapses at nearby locations on the dendrite (black circles).

Most of the synaptic inputs that reach a neuron arrive on its dendrites. How these signals are integrated and finally transformed into action potentials is of central importance for understanding neuronal information processing (Spruston et al., 2016). Dendrites can support information processing at multiple spatial scales (Figure 1). On the local scale, i.e., the fine-scale organization of synapses over tens of microns, dendrites affect information processing by organizing synapses with similar properties into *synaptic clusters* that boost a neuron’s computational capacity through nonlinear integration (Kastellakis et al., 2015; Mel, 1992, 1993; Poirazi et al., 2003; Ujfalussy and Makara, 2020; Wilson et al., 2016). On the level of individual branches, dendrites exhibit *dendritic maps* in which the tuning of synapses (or synaptic clusters) varies systematically across the dendritic tree (Bollmann and Engert, 2009; Iacaruso et al., 2017; Jia et al., 2014; Podgorski et al., 2021; Wilson et al., 2016). Finally, the dendrite’s branching structure determines the extent to which synaptic inputs influence the soma (Ferrante et al., 2013; Jaffe and Carnevale, 1999; Tzilivaki et al., 2019; Vetter et al., 2001).

There is accumulating evidence that a substantial amount of the dendritic organization across these scales emerges already during early development (Kirchner and Gjorgjieva, 2021; Kleindienst et al., 2011; Lee et al., 2016; Niculescu et al., 2018; Takahashi et al., 2012; Winnubst et al., 2015). This early emergence is particularly noteworthy as neural activity during these early phases primarily arises spontaneously (Leighton and Lohmann, 2016), raising the question of how the brain can precisely arrange synaptic inputs across scales without sensory input. Investigating this question reveals essential facts about the mechanisms of brain development and provides a valuable perspective on how the adult brain works.

### Synaptic clustering

**Figure 2:**
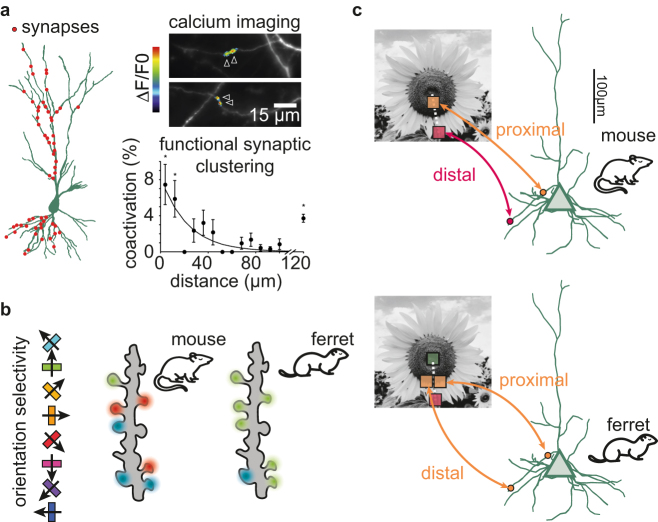
Species-specific local and global organization of excitatory synapses. **(a)** Left: reconstruction of a CA3 pyramidal neuron with synaptic inputs indicated as red circles. Redrawn from ref (Kleindienst et al., 2011). Top right: calcium activity in clustered synaptic inputs in developing CA3 pyramidal cell dendrites. Redrawn from ref (Niculescu et al., 2018). Bottom right: synaptic coactivation as a function of intersynaptic distance. Redrawn from ref (Kleindienst et al., 2011). **(b)** Illustration of qualitatively different types of clustering in mouse and the ferret: the ferret shows clustering according to orientation, whereas the mouse does not and instead shows clustering according to receptive field overlap (Iacaruso et al., 2017; Wilson et al., 2016). **(c)** Illustration of a retinotopically organized dendritic map observed in the mouse (top) but not in the ferret (bottom).

In the case of local organization, synapses onto mouse pyramidal neurons arrange into clusters during early postnatal development (Kleindienst et al., 2011; Niculescu et al., 2018; Takahashi et al., 2012; Winnubst et al., 2015) (Figure 2a). Clustering refines over development (Takahashi et al., 2012) and cannot form when spontaneous activity is absent (Kleindienst et al., 2011; Takahashi et al., 2012). Further, experiments blocking individual molecules and their receptors implicate a family of signaling molecules called *neurotrophic factors* in this process (Kutsarova et al., 2021; Niculescu et al., 2018; Winnubst et al., 2015). Computational modeling demonstrates that the interactions between these neurotrophic factors effectively implement a local plasticity rule that can generate clustering (Kirchner and Gjorgjieva, 2021), where poorly synchronized synapses weaken and well-synchronized synapses stabilize (Niculescu et al., 2018; Winnubst et al., 2015). As the activity becomes driven by the senses and the animal encounters more complex situations, the substrate of plasticity becomes more complex over development. While the prevalence of the relevant neurotrophic factors decreases with age (Yang et al., 2009), there is evidence that the local plasticity rule also applies in the adult animal, possibly implemented by different sets of interacting molecules (El-Boustani et al., 2018; Harward et al., 2016; Hedrick et al., 2016; Oh et al., 2015; Tazerart et al., 2020). This parallel hints at the exciting possibility that the underlying principles of synaptic plasticity remain unchanged and form a foundation for more versatile plasticity during adult life (Ganguly and Poo, 2013; Lohmann and Kessels, 2014).

While synaptic clustering appears to be near-ubiquitous across brain areas and species (Adoff et al., 2021; Ashaber et al., 2021; Frank et al., 2018; Fu et al., 2012; Gökçe et al., 2016; Iacaruso et al., 2017; Ju et al., 2020; Kerlin et al., 2019; Kim et al., 2021; Kleindienst et al., 2011; Lee et al., 2019; McBride et al., 2008; Niculescu et al., 2018; Podgorski et al., 2021; Scholl et al., 2017; Takahashi et al., 2012; Wilson et al., 2016; Winnubst et al., 2015), there is striking variability in the qualitative characteristics of clusters. The receptive field of a synapse – the sensory feature encoded by the synaptic input – can be used to describe the properties of clusters. For example, synaptic clusters in the ferret or macaque visual cortex tend to have receptive fields that share a preference for moving gratings of the same orientation (Scholl et al., 2017; Wilson et al., 2016). This shared orientation preference is not present in the mouse visual cortex, where instead, synapses with spatially overlapping receptive fields tend to form a cluster (Iacaruso et al., 2017; Jia et al., 2010) (Figure 2b). Computational modeling demonstrates that the differences between the mouse, ferret, and macaque visual cortex might result from two simple factors: the anatomical size of the retina and the anatomical size of the visual cortex (Kirchner and Gjorgjieva, 2021; Scholl et al., 2017).

Thus, central aspects of the local synaptic organization can emerge without sensory stimulation, suggesting that development might equip dendrites with fundamental building blocks such as feature selectivity from which other functional properties derive in adulthood. Computational modeling stands out as a handy tool for investigating this hypothesis, as longitudinal experiments that monitor synaptic organization and function across development and into adulthood (Witvliet et al., 2021) are currently technically infeasible.

### Dendritic maps

Beyond the fine-scale organization over tens of microns, there is also accumulating evidence for synaptic organization on the level of entire dendritic branches (hundreds of microns). Concretely, synapses do not only cluster locally but also tend to organize along the entire dendritic tree, according to their function (Bollmann and Engert, 2009; El-Boustani et al., 2018; Iacaruso et al., 2017; Jia et al., 2014; Kerlin et al., 2019; Podgorski et al., 2021; Wilson et al., 2016). One striking example of this can be observed in the *Xenopus* tadpole tectum (Bollmann and Engert, 2009) and in the mouse visual cortex (El-Boustani et al., 2018; Iacaruso et al., 2017) where synapses are arranged retinotopically, i.e., proximal (distal) synapses tend to respond to stimulation of central (peripheral) locations in visual space (Figure 2c). We note, however, that the existing experimental data in the mouse and ferret visual cortex do not provide information about the relationship between synapse proximity and location on basal versus apical dendrites. A similar synaptic organization can be observed in the mouse barrel cortex, where proximal (distal) synapses tend to respond (not respond) to stimulation of the primary whisker of the corresponding barrel (Jia et al., 2014; Schoonover et al., 2014), and in the hippocampus, where individual branches respond to specific locations in space (Rashid et al., 2020). We term this type of global organization *dendritic maps* (Kirchner and Gjorgjieva, 2021) to highlight the similarity with *cortical maps* (White and Fitzpatrick, 2007). In the ferret visual cortex, this retinotopic organization is markedly absent (Scholl et al., 2017) (Figure 2c), and instead, synapses on the same dendritic branch tend to share the same preference for oriented gratings (Wilson et al., 2016).

While experimental evidence for dendritic maps abounds, their possible function is mostly unclear. One explanation for why different dendritic branches receive different inputs is that this separation allows the soma to weigh the inputs according to their reliability, enabling Bayes-optimal integration (Jordan et al., 2021). Alternatively, different dendritic branches might be gated on or off in a context-dependent fashion (Yang et al., 2016), allowing more powerful dendritic computations (Poirazi et al., 2003) and modifying only a subset of synapses while retaining the rest (Cichon and Gan, 2015; Sezener et al., 2021; Yaeger et al., 2019). A third hypothesis is that the feature selectivity of different synapses matches commonly co-occurring features in complex sensory inputs, allowing the neuron to perform more efficient feature detection (Hiratani and Fukai, 2018; Iacaruso et al., 2017; Kirchner and Gjorgjieva, 2021). Finally, the ability of dendrites to segregate feed forward and feedback information into different compartments (Larkum et al., 2009; Takahashi et al., 2016) might provide a biological substrate for determining the contribution of individual inputs to the outcome of a computation, known as the credit assignment problem (Guerguiev et al., 2017; Richards and Lillicrap, 2019).

However, recent experiments have revealed that our understanding of dendritic computations is still limited. The somatic receptive field seems to be derived from only a handful of powerful synaptic connections (Cossell et al., 2015). Indeed, removing the entire basal or apical dendrites *in vivo* does not affect the functional selectivity of the soma (Park et al., 2019). Also, large dendritic events *in vivo* overwhelmingly co-occur with somatic events in typical recording setups (Beaulieu-Laroche et al., 2019; Francioni et al., 2019; Kerlin et al., 2019) and only become substantially decoupled when the animal is allowed to move freely (Voigts and Harnett, 2020). These observations indicate that proper investigation of structured and diverse synaptic input across the entire dendritic tree might require a richer set of stimuli (Laboy-Juárez et al., 2019) that engages the full cognitive potential of the animal.

How might dendritic maps be established? In contrast to synaptic clustering, there is only limited experimental evidence of the presence of dendritic maps in early development (Bollmann and Engert, 2009). Computational modeling suggests that local, structural plasticity in conjunction with an attenuating back-propagating action potential is sufficient to produce species-specific dendritic maps (Kirchner and Gjorgjieva, 2021). Alternatively, (Hiratani and Fukai, 2018) propose that dendritic maps might also arise during development from a combination of plasticity that depends on the location of the synapse on the dendritic tree (Froemke et al., 2005; Letzkus et al., 2006; Weber et al., 2016) and structural synaptic rewiring (Mel, 1992). Finally, recent experimental data demonstrate that dendritic growth is affected by synaptic activity (Podgorski et al., 2021), suggesting that changes to dendritic morphology might facilitate dendritic map formation. Since early development is characterized by the establishment of dendritic morphology (Richards et al., 2020) and by repeated and rapid synapse turnover (Holtmaat and Svoboda, 2009; Maletic-Savatic et al., 1999), it appears as a particularly opportune time for the formation of dendritic maps.

In summary, there is accumulating evidence that dendrites exhibit dendritic maps, i.e., topographically organized synaptic inputs across the entire dendritic tree. These dendritic maps exhibit substantial qualitative variability across areas and species. While their function is still unclear, computational models generate several testable predictions and provide hypotheses that can guide the direction of future experimental research.

## Organization of inhibitory synapses

Most studies on synaptic organization focus on excitatory synapses. This focus is traditionally attributable to the relative abundance of glutamatergic synapses and the better availability of markers for glutamatergic synapses (Chen et al., 2012). Even though inhibitory synapses, which represent 12% of all dendritic synapses (Iascone et al., 2020), have been relatively neglected by comparison, they play an essential role in neural information processing (Boivin and Nedivi, 2018).

### Local balance of excitatory and inhibitory synapses

**Figure 3:**
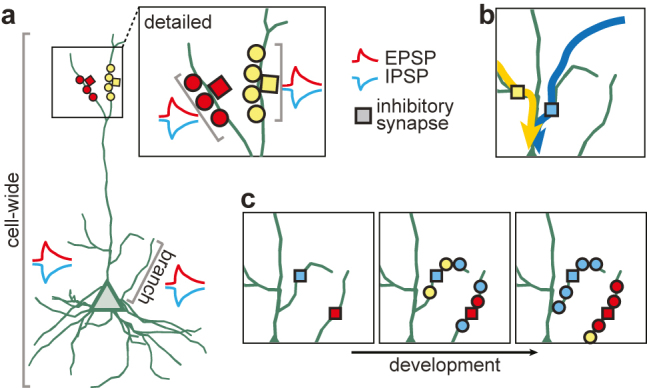
Function and origin of locally balanced excitation and inhibition. **(a)** Illustration of the balance of excitation and inhibition at different spatial scales. Balance might exist cell-wide (excitation and inhibition matched at the soma), on individual branches, or local stretches of the dendrite (detailed balance, inset, top right). **(b)** Inhibitory synapses (colored squares) might gate dendritic signals by selectively inhibiting some branches but not others (Boivin and Nedivi, 2018). Yellow and blue arrows indicate incoming signals from two different branches. **(c)** Model of the emergence of excitatory and inhibitory balance over development, based on experiments in the mouse retina (Bleckert et al., 2013; Johnson et al., 2003; Soto et al., 2011). Inhibitory synapses form first (left) and provide a scaffold around which excitatory synapses organize (middle). Structural and functional plasticity rearranges excitatory synapses to establish a detailed dendritic balance (right).

The balance of excitation and inhibition is a characteristic feature of cortical dynamics (Okun and Lampl, 2008). Still, it is an open question on just how *detailed* this balance is (Hennequin et al., 2017): does the balance extend to the dendritic tree, individual branches, or even local stretches on the dendrite (Figure 3a)? These questions are the center of new experimental studies on inhibitory synapse organization and dynamics.

On the local scale, inhibitory synapses exhibit a substantial amount of organization (Chen et al., 2012; Iascone et al., 2020; Liu, 2004; Villa et al., 2016). In contrast to excitatory synapses, which primarily reside on dendritic spines, inhibitory synapses can be found both on spines and the dendritic shaft (Iascone et al., 2020). The density of inhibitory synapses closely tracks the density of excitatory synapses (Iascone et al., 2020), and 25–30% of all inhibitory synapses share a dendritic spine with an excitatory synapse (Chen et al., 2012; Iascone et al., 2020), an arrangement called *dually innervated spines*. Excitatory synapses on dually innervated spines are extraordinarily stable and experience almost no turnover (Villa et al., 2016). By contrast, inhibitory synapses on dually innervated spines experience increased remodeling, repeatedly appearing and disappearing on the same spine (Villa et al., 2016). Experimental stimulation of clustered excitatory synapses triggers the *de novo* formation of inhibitory synapses (Hu et al., 2019). Also, during normal visual experience, whenever excitatory synapses are stable, nearby inhibitory synapses within 10 μm are also stable (Chen et al., 2012). Finally, experiments in a hippocampal culture provide evidence for a push–pull plasticity mechanism that can carefully balance excitatory and inhibitory strength (Liu, 2004). Thus, a detailed balance of excitation and inhibition on local stretches of dendrites appears entirely consistent with the experimental data.

Based on their location on specific parts of the dendritic tree, inhibitory synapses can effectively shape dendritic signal detection and integration by restricting both voltage and calcium signaling of excitatory synapses (Boivin and Nedivi, 2018; Higley, 2014; Liu, 2004) (Figure 3b). Computational modeling proposes several possible benefits of this property of inhibitory synapses. Consistent with experiments (Wang and Maffei, 2014), modulating local inhibition can change the shape of the learning rule for excitatory synapses (Agnes and Vogels, 2021; Hiratani and Fukai, 2017; Mikulasch et al., 2021). Based on the relative timing and strength of the inhibitory input, potentiation and depression of excitatory synapses can be attenuated (Agnes and Vogels, 2021) or even fully inverted (Hiratani and Fukai, 2017; Mikulasch et al., 2021). This inhibitory control over excitatory plasticity might enable the recalibration of selectivity of excitatory synapses in the visual cortex when input from one eye is lost (Hiratani and Fukai, 2017). Alternatively, local inhibition can help to avoid redundancies in the neural code and lead to more efficient and diverse feature representations (Mikulasch et al., 2021). Finally, gating excitatory plasticity through local inhibition can allow for the rapid reorganization of excitatory synapses during periods of disinhibition, while keeping excitatory organization stable (Agnes and Vogels, 2021; Sezener et al., 2021).

How a highly detailed balance of excitation and inhibition emerges in the cortex is still an open question, but studying the emergence of detailed balance in the mouse retina (Bleckert et al., 2013; Jain et al., 2020; Johnson et al., 2003; Soto et al., 2011) might provide important insights. In the retina, inhibitory synapses form slightly before excitatory synapses (Johnson et al., 2003); the density of both types of synapses increases gradually until eye opening (Soto et al., 2011); and excitatory synapses are more likely to form nearby inhibitory synapses (Bleckert et al., 2013). These constraints postulate a model in which inhibitory synapses form a stable backbone around which excitatory synapses cluster (Kirchner and Gjorgjieva, 2021). Once the density of excitatory and inhibitory synapses has stabilized, a local plasticity rule can further rearrange synapses and establish synaptic clusters to enable flexible computations and efficient learning (Agnes and Vogels, 2021; Hiratani and Fukai, 2017; Kirchner and Gjorgjieva, 2021; Mikulasch et al., 2021) (Figure 3c).

While less is known about the organization of inhibitory synapses than of excitatory synapses, recent experiments and modeling studies paint a picture in which both types of synapses interact closely. Inhibitory synapses can exercise tight control over excitatory activity and plasticity, allowing for more flexible and diverse neural computations. During development, inhibitory synapses might form a backbone that substantially constrains and guides the emergence of excitatory synapse organization.

## Discussion

The precise organization of synapses across the dendritic tree makes us hopeful that the daunting amount of detail present in biological dendrites as the branches that enable neurons to connect and transmit information might eventually lead to the discovery of unifying principles. Synaptic clustering, dendritic maps, and detailed excitatory–inhibitory balance all share the hallmark features of organization of the cortex at large: nearby neurons tend to share functional properties (Dombeck et al., 2009), neurons across the cortical sheet arrange into cortical maps (White and Fitzpatrick, 2007), and excitatory and inhibitory activity at the level of the soma is balanced (Okun and Lampl, 2008). We are excited to see if further parallels will emerge as experimental methods become more powerful (Podgorski et al., 2021).

While we focused on the organization of synapses on dendrites, conversely, dendrites’ shape is affected by the availability of suitable synaptic partners (Niell et al., 2004; Podgorski et al., 2021; Stuart et al., 2016). As a consequence, a mature dendrite’s shape might not only affect its possible inputs and thereby its computational capacities but also be the result of activity-dependent plasticity processes during early development. A time-lapsed optical imaging of dendrite growth during spontaneous activity or in conjunction with sensory stimulation (Podgorski et al., 2021) will be able to ascertain the degree to which activity affects dendrite growth and vice versa.

A wide range of further distinctions is possible beyond the broad separation of synapses into excitatory and inhibitory. Inhibitory interneurons fall into several genetically characterized subtypes, which differ in their biophysical properties and subcellular specificity (Tremblay et al., 2016). While fast-spiking, parvalbumin-positive interneurons tend to form synapses onto the soma and the proximal dendrite, other interneuron types prefer the distal basal or the apical tuft. While research on circuit implications of interneuron diversity is well underway, functional implications of different interneuron subtypes on different parts of the dendrite are only just shifting into focus (Vercruysse et al., 2021).
